# *Helicobacter pylori* infection reduces disease severity in an experimental model of multiple sclerosis

**DOI:** 10.3389/fmicb.2015.00052

**Published:** 2015-02-13

**Authors:** Katherine W. Cook, James Crooks, Khiyam Hussain, Kate O’Brien, Manjit Braitch, Huner Kareem, Cris S. Constantinescu, Karen Robinson, Bruno Gran

**Affiliations:** ^1^Nottingham Digestive Diseases Biomedical Research Unit, Centre for Biomolecular Sciences, University of Nottingham School of MedicineNottingham, UK; ^2^Clinical Neurology Research Group, Division of Clinical Neuroscience, University of Nottingham School of MedicineNottingham, UK

**Keywords:** *Helicobacter pylori*, multiple sclerosis, experimental autoimmune encephalomyelitis, T-helper subsets

## Abstract

Recent research has demonstrated that infection with the bacterial pathogen *Helicobacter pylori* is less common amongst patients with multiple sclerosis (MS), an inflammatory demyelinating disease of the central nervous system (CNS). We aimed to compare the prevalence of *H. pylori* amongst MS patients and healthy controls, and also investigated the impact of this infection on an animal model for MS, experimental autoimmune encephalomyelitis (EAE). The *H. pylori* status of 71 MS patients and 42 healthy controls was determined by serology. Groups of C57BL/6 mice were infected with *H. pylori*, or given diluent alone as a placebo, prior to inducing EAE. Clinical scores were assessed for all mice, and spleens and spinal cord tissue were harvested. CD4^+^ T cell subsets were quantified by flow cytometry, and T cell proliferation assays were performed. In MS patients the seroprevalence of *H. pylori* was half that of healthy controls (*p* = 0.018). Over three independent experiments, prior *H. pylori* infection had a moderate effect in reducing the severity of EAE (*p* = 0.012). In line with this, the antigen-specific T cell proliferative responses of infected animals were significantly reduced (*p* = 0.001), and there was a fourfold reduction in the number of CD4^+^ cells in the CNS. CD4^+^ populations in both the CNS and the spleens of infected mice also contained greatly reduced proportions of IFNγ^+^, IL-17^+^, T-bet^+^, and RORγt^+^ cells, but the proportions of Foxp3^+^ cells were equivalent. There were no differences in the frequency of splenic CD4^+^cells expressing markers of apoptosis between infected and uninfected animals. *H. pylori* was less prevalent amongst MS patients. In mice, the infection exerted some protection against EAE, inhibiting both Th1 and Th17 responses. This could not be explained by the presence of increased numbers of Foxp3^+^ regulatory T cells, or T cell apoptosis. This is the first direct experimental evidence showing that *H. pylori* may provide protection against inflammatory demyelination in the CNS.

## INTRODUCTION

*Helicobacter pylori* is a Gram negative bacterium that usually establishes lifelong colonization of the human stomach from early childhood. Approximately 10–15% of those infected develop symptomatic disease, including gastric or duodenal ulcers and gastric cancer (Atherton, 2006). In the vast majority of cases, however, colonization leads to asymptomatic chronic gastritis, with increased infiltration of neutrophils, dendritic cells (DCs), macrophages, natural killer (NK) cells, and lymphocytes into the gastric mucosa (reviewed in Robinson and Atherton, 2010; Koch et al., 2013). There is increased abundance of pro-inflammatory T-helper 1 (Th1) and Th17 subsets, as well as anti-inflammatory regulatory T cells (Tregs) (Lundgren et al., 2005; Robinson et al., 2008; Serrano et al., 2013). Infected individuals without gastro-duodenal disease tend to have a more robust Treg response, which may also provide protection against extra-gastric conditions such as asthma, allergy, and inflammatory bowel disease (Kao et al., 2010; Arnold et al., 2011, 2012; Wang et al., 2013; Amberbir et al., 2014).

There have been a number of cross-sectional epidemiological studies reporting a lower prevalence of *H. pylori* amongst patients with multiple sclerosis (MS) (Wender, 2003; Li et al., 2007; Mohebi et al., 2013; Yoshimura et al., 2014). A recent case control study in Iran also reported that neurological disability was lower in *H. pylori-*seropositive than in seronegative MS patients (Mohebi et al., 2013), in agreement with a previous study in a Japanese MS patient cohort (Li et al., 2007). In contrast, some other studies have failed to find any association between *H. pylori* infection and MS (Danese et al., 2000), and there is strong serological evidence to support a positive association between *H. pylori* and neuromyelitis optica (NMO), an antibody-mediated, severe variant of MS that involves the spinal cord and the optic nerves (reviewed by Smyk et al., 2014).

The incidence of autoimmune diseases has been increasing worldwide over the last few decades and their prevalence has been linked to decreased exposure to microbial infections including *H. pylori* (Okada et al., 2010). MS is an inflammatory demyelinating immune-mediated disorder which affects the central nervous system (CNS). Development of autoreactive T cell responses against CNS-derived antigens leads to infiltration of Th1 and Th17 cells into the CNS (Goverman, 2009; Baker et al., 2011), resulting in damage to the myelin sheath of neural axons, inflammation, and neurodegeneration (Frohman et al., 2006). Studies have shown that the balance between pro-inflammatory Th1 and Th17 responses and anti-inflammatory Treg responses, either in terms of numbers or functional activity, are important in MS development and progression (Edstrom et al., 2011; Sellebjerg et al., 2012). We previously showed that patients infected with *H. pylori* have elevated Treg populations in their peripheral blood (Cook et al., 2014). We hypothesized that *H. pylori* protects against MS via the stimulation of increased numbers of Tregs, which enter the CNS to suppress the effector T cell-mediated inflammation and damage.

So far there is very little evidence to conclusively evaluate the links between MS and *H. pylori.* No data have been published on the impact of *H. pylori* eradication therapy on MS, and only one animal model study has been reported so far (Boziki et al., 2012). This showed that co-administration of heat killed *H. pylori* bacteria, with the injected doses of myelin oligodendrocyte glycoprotein (MOG) peptide and adjuvant used to induce EAE, completely inhibited EAE development. However, there was no attempt to evaluate the mechanism.

The current study aimed to determine the effect of *H. pylori* infection on the development and severity of EAE. C57BL/6 mice were infected with *H. pylori* or given a placebo by oral gavage 3 weeks prior to EAE induction treatment. *H. pylori* infected mice had significantly decreased EAE clinical scores, accompanied by markedly lower frequencies of CD4^+^ and CD8^+^ cells in the spinal cord. In the spleen, the proportions of Th1 and Th17 cells amongst the CD4^+^ population were significantly diminished but there were no differences in the frequency of Foxp3^+^ cells. This trend was also found in the CNS. Protection therefore did not appear to be mediated via increased Foxp3^+^ Treg infiltration of the CNS.

## MATERIALS AND METHODS

### HUMAN CLINICAL SAMPLES

Venous blood samples were collected from 71 MS patients (20 male, 51 female; mean ± SD age 53 ± 10; 48 with relapsing-remitting MS, 19 with secondary progressive MS and 4 with primary progressive MS) and 42 age and gender matched healthy controls (15 male, 27 female; mean ± SD age 50 ± 11) at the Queen’s Medical Centre, Nottingham, UK, with informed written consent and approval from the Nottingham Research Ethics Committee 2. Seventeen patients were on disease modifying treatment (10 interferon; two copaxone; one daclizumab; one fingolimod; one azathioprine). Serum was separated, aliquoted and stored at -80°C. *H. pylori* status was determined using a Biohit *H. pylori* IgG ELISA kit (Biohit Healthcare Ltd., Cheshire, UK), according to the manufacturer’s instructions.

### ANIMAL EXPERIMENTS

All animal experiments were approved by the University Animal Welfare and Ethical Review Body and performed in accordance with the UK Home Office License regulations, under Project License 40/3676. 6 week old female C57BL/6 mice were infected by oral gavage on three alternate days, with doses of 1 × 10^9^ *H. pylori* strain SS1 in 100 μL *Brucella* broth (Oxoid Ltd., Basingstoke, UK), as previously described (Winter et al., 2014). *H. pylori* colonization was confirmed at 3 weeks post inoculation, by conducting *H. pylori* stool antigen tests on freshly collected fecal pellets using the Premier Platinum HpSA® PLUS kit (Meridian Bioscience Inc., Cincinnati, OH, USA). At 3 weeks post infection, EAE induction treatment was commenced: mice were immunized via sub-cutaneous injection at two sites with MOG peptide MOG_35-55_ (Cambridge Research Biochemicals; 275 μg/mouse) in 0.2 ml incomplete Freund’s adjuvant (DIFCO, Becton Dickinson & Company, Franklin Lakes, NJ, USA), containing 4 mg/ml *Mycobacterium tuberculosis* H37 Ra (DIFCO; O’Brien et al., 2010). An intraperitoneal injection of 200 ng pertussis toxin (List Biological Laboratories Inc., Campbell, CA, USA) was also administered on the same day, with a second subsequent dose 2 days later. Mice were weighed and scored at least once daily in a blinded manner, according to a published clinical scoring scale (O’Brien et al., 2010): 0-healthy, 1-flaccid tail, 2-impaired righting reflex and/or impaired gait, 3-partial hind-leg paralysis, 4-total hind-leg paralysis, 5-any sign of front-leg paralysis, and 6-moribund/dead. Mice were monitored and humanely euthanized at the end of the experiment, or if symptoms reached the authorized endpoint (weight loss reaching 25%, a score of 4 lasting up to 5 days, or a score above 4 at any point during the study).

For assessment of *H. pylori* effects on markers of T cell and Foxp3^+^ cell apoptosis, working under Home Office Project License 40/3399, Foxp3-green fluorescent protein (GFP) C57/BL6 mice (JAX strain B6.Cg-Foxp3^tm2(EGFP)Tch^/J) (Haribhai et al., 2007) were infected by oral gavage on three alternate days with doses of 1 × 10^9^ *H. pylori* and killed at 3 weeks post infection as above.

### CELL ISOLATION AND ANALYSIS BY FLOW CYTOMETRY

Mice were humanely killed 3 weeks after the start of EAE induction treatment and perfused with PBS prior to removal of tissues. Spleens were collected into culture medium RPMI 1640/10% fetal calf serum/100 U/mL penicillin G/100 μg/mL streptomycin sulfate (Sigma–Aldrich, Poole, UK). Individual spleens were rubbed through sterile disposable 40 μm cell strainers (Fisher Scientific UK Ltd., Loughborough, UK), and the cells treated with Red Blood Cell Lysis Buffer (Sigma–Aldrich), washed and resuspended at 1 × 10^6^/ml in culture medium. Spinal cords were removed from the mice, disrupted through a 100 μm cell strainer (Fisher) and washed with PBS. The pooled spinal cord cells from each treatment group were fractionated in a 60/30% Percoll gradient (GE Healthcare, Buckinghamshire, UK), by centrifugation at 300 ×*g* for 20 min. Mononuclear cells were harvested from the interface, washed and resuspended at 1 × 10^6^/ml in culture medium.

1 ml of cells was aliquotted into sterile 12 × 75 mm culture tubes (Elkay Laboratory Products UK Ltd., Basingstoke, UK). For direct analysis of cell surface markers and transcription factors expressed by Th1, Th17, and Treg subsets (T-bet, RORγt and Foxp3, respectively), the cells were stained immediately. CD4^+^ cells expressing the signature Th1 and Th17 cytokines IFNγ and IL-17A were quantified after stimulation with phorbol myristate acetate (PMA) and ionomycin (Robinson et al., 2008). As a negative control, medium alone was added. The tubes were incubated at 37°C in an atmosphere of 5% carbon dioxide, and brefeldin A (Sigma–Aldrich) was added to a final concentration of 10 μg/ml after the first hour of a 6-h long incubation period.

As previously described (Cook et al., 2014), extracellular staining using fluorochrome-conjugated anti-CD4-phycoerythrin-Texas-Red (ECD; Beckman Coulter UK Ltd., Buckinghamshire, UK) and anti-CD8-phycoerythrin-cyanin 5 (PC5; eBioscience Ltd., Hatfield, UK) was carried out before cells were fixed in 0.5% formaldehyde (Sigma–Aldrich). For intracellular markers, the cells were permeabilised with FOXP3 Perm Buffer (BioLegend, London, UK), before staining with anti-Foxp3-Alexa Fluor 488 (A488; BioLegend), anti-T-bet-Alexa Fluor 647 (A647; eBioscience), anti-RORγt-phycoerythrin (PE; eBioscience), or anti-IL-17A-PE (BioLegend) and anti-IFNγ-A488 (BD Pharmingen, Oxford, UK), or the appropriate isotype controls. Data on 200,000 events per tube was acquired using an FC500 flow cytometer (Beckman Coulter Cytomics). Analysis was performed using Weasel version 3.0 (http://www.wehi.edu.au/faculty/advanced_research_technologies/flow_cytometry/weasel_for_flow_cytometry_data_analysis), with respect to isotype controls and fluorescence-minus-one controls.

### PROLIFERATION ASSAY

As described previously (O’Brien et al., 2010), 4 × 10^5^ spleen cells in culture medium (0.2 ml per well) were plated in 96 well U-bottom Corning Costar cell culture plates (Sigma–Aldrich). MOG_35-55_ peptide was added to final concentrations of 1, 10, or 100 μg/ml. As a positive control, 1 μg/ml anti-CD3 and anti-CD28 antibodies (Beckman Coulter) were added. The equivalent volume of medium was added as a negative control. Cells were cultured for 72 h at 37°C with 5% carbon dioxide, pulsed with 1 μCi [^3^H]thymidine (Perkin Elmer, Cambridge, UK) per well, and cultured for a further 16 h. The cells were harvested onto glass fiber filter mats using a Packard harvester, and the plates were left to dry overnight. 25 μl of Microscint scintillation fluid (Perkin Elmer) was then added to each well before being assessed for thymidine incorporation using a liquid scintillation β counter (Top Count, Microplate Scintillation Counter; Packard, UK). Cell proliferation was recorded in counts per minute (CPM).

### T CELL APOPTOSIS

Groups of 4 C57BL/6 Foxp3-GFP reporter mice were infected with *H. pylori* or given oral doses of *Brucella* broth as a placebo. Three weeks later, the mice were killed, splenocytes were harvested and immediately stained with fluorochrome-conjugated antibodies [anti-CD4-ECD (Beckman Coulter), anti-active caspase-3-PE (BD Pharmingen), anti-Fas-PC7 (BD Pharmingen), anti-FasL-PE (BD Pharmingen)], and propidium iodide (PI; Invitrogen), as markers for apoptosis or cell death. The cells were then analyzed by flow cytometry, detecting GFP^+^ events as a marker for Foxp3. Stimulation for 3 h with 5 μM camptothecin (MP Biomedicals, Santa Ana, CA, USA) as a positive control inducer of apoptosis, resulted in >70% of CD4^+^ events staining positive for active caspase-3.

### STATISTICAL ANALYSIS

Statistical analyses were carried out using Prism 6.00 (GraphPad, Software CA, USA). A *p-*value < 0.05 was taken as significant. A Chi-squared test was used to compare the prevalence of *H. pylori* infection amongst MS patients and healthy controls. A Mann–Whitney *U*-test was used to compare clinical scores and immunological parameters between treatment groups in mouse experiments.

## RESULTS

### FEWER MS PATIENTS WERE *H. pylori*-POSITIVE COMPARED WITH HEALTHY CONTROLS

Serum samples were collected from MS patients and matched healthy controls at the Queen’s Medical Centre, Nottingham, UK. *H. pylori* infection status was determined by serology. Of the MS patients, 21.1% were infected with *H. pylori* (15/71) compared with 42.9% (14/42) of the healthy controls (Chi squared test, *p* = 0.018). This shows that patients with MS were half as likely to have a *H. pylori* infection. There were no differences in the proportion of patients receiving disease modifying treatment between *H. pylori* positive and negative patients; the distribution of MS clinical subtypes, or the gender or age showed no differences between *H. pylori* positive and negative patients. In order to investigate whether the results could be due to a direct protective effect we then performed infection experiments in the well-established mouse model for MS, EAE.

### EAE CLINICAL SCORES WERE REDUCED IN MICE INFECTED WITH *H. pylori*

**FIGURE 1:**
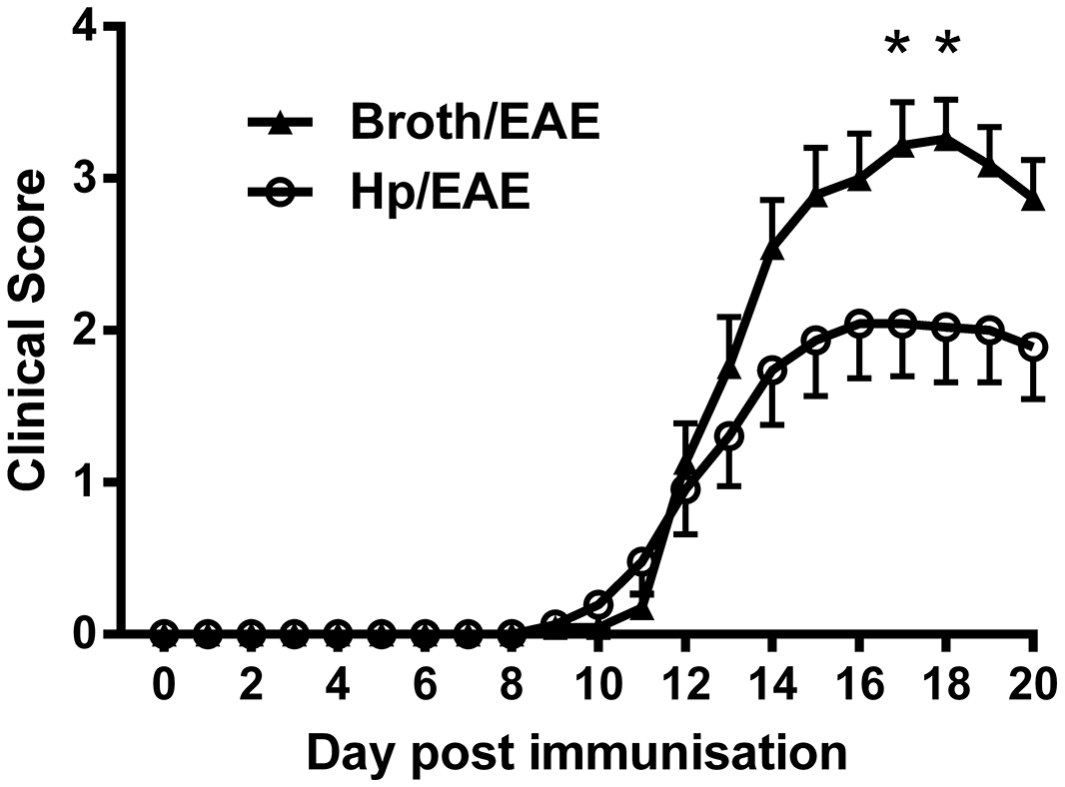
**Clinical scores from mice inoculated with *Helicobacter pylori* or broth before experimental autoimmune encephalomyelitis (EAE) induction.** Mice were infected with *H. pylori* (◯), or given a placebo broth dose (▲). After 3 weeks EAE induction treatment with MOG peptide was administered (day 0). Clinical scores were recorded daily. Graph shows the mean clinical scores and SEM for each group of six mice, from three independent experiments. *p = 0.05.

Three independent experiments were performed in mice, in order to investigate the effect of *H. pylori* infection on EAE. Mice were given oral doses of 1 × 10^9^ *H. pylori* (Hp/EAE group) or plain *Brucella* broth as a placebo (Broth/EAE group). After 3 weeks, EAE was induced in all mice, the animals were closely monitored and clinical scores recorded daily (**Figure 1**). The cumulative scores, maximal scores, and time to symptomatic EAE onset were calculated (**Table 1**).

**Table 1:** Key parameters from experimental autoimmune encephalomyelitis (EAE) clinical scores of *Helicobacter pylori-*infected and broth placebo-treated mice.

	Cumulative score	*p*-value	Maximal score	*p*-value	Day of onset	*p*-value
Broth/EAE	22.59 ± 2.00	0.103	3.54 ± 0.24	0.012	12.89 ± 0.59	0.715
Hp/EAE	15.73 ± 2.85		2.39 ± 0.35		12.48 ± 0.37	

*Animals were scored daily for clinical signs of disease in line with published criteria. Data represents mean ± SEM. p-values were calculated using Mann–Whitney U-tests.*

Overall, clinical scores were reduced in mice infected with *H. pylori* prior to EAE induction. At both day 17 and day 18 there was a significant difference in the mean clinical scores observed between Hp/EAE and Broth/EAE groups (*p* = 0.05). In addition, the mean maximal score in the Broth/EAE group was 3.54 compared to 2.39 in the Hp/EAE group (*p* = 0.012). There was a trend for a reduced cumulative score in the *H. pylori* infected animals, although this did not reach statistical significance (*p* = 0.103). The time to EAE onset was similar in both treatment groups (**Table 1**).

### MOG PEPTIDE-SPECIFIC PROLIFERATION WAS REDUCED IN SPLENOCYTES FROM *H. pylori*-INFECTED MICE

**FIGURE 2:**
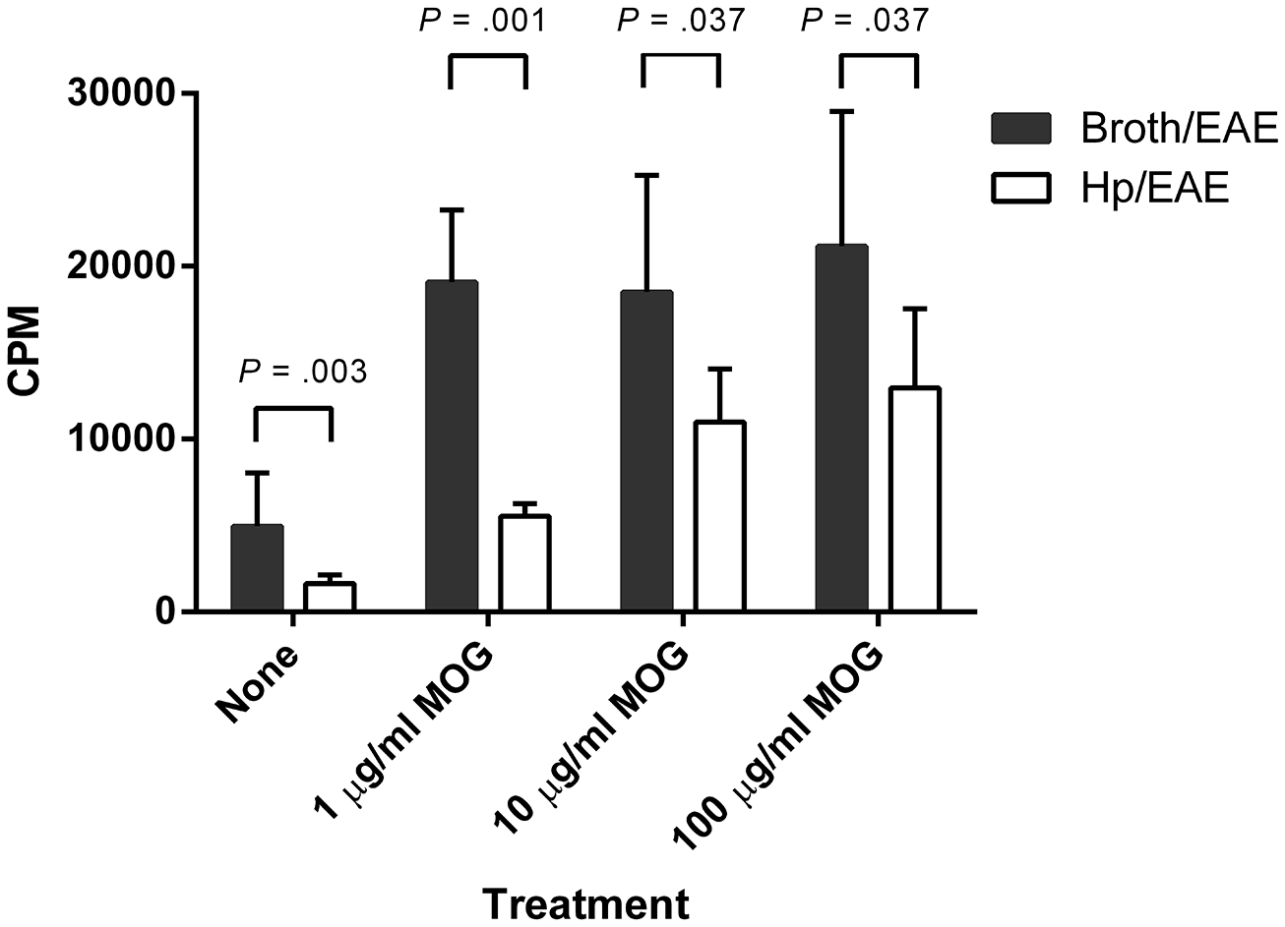
**Proliferation of splenocytes in response to stimulation by MOG_**35**-**55**_ peptide.** Mice were infected with *H. pylori* (Hp/EAE), or given a placebo dose (Broth/EAE). After 3 weeks, EAE induction treatment with MOG_35-55_ peptide was administered. Splenocytes were isolated from mice after a further 3 weeks. Splenocytes were stimulated with MOG_35-55_ peptide and incorporation of [^3^H]thymidine was used to measure the proliferative response in counts per minute (CPM). The median CPMs following stimulation with anti-CD3/28 antibodies were 118946 and 57633 for the Broth/EAE and Hp/EAE groups, respectively. Bars depict the mean CPM for mice in each group; error bars represent SD. *p*-values were calculated using Mann–Whitney *U-*tests.

We then assessed whether *H. pylori* infection had an impact on the MOG-specific T cell response generated. Splenocytes isolated from the Hp/EAE mice made a significantly impaired MOG-specific proliferation response (**Figure 2**). With 1 μg/ml MOG, the splenocytes from Hp/EAE mice proliferated threefold less than the Broth/EAE control group (*p* = 0.001), and there were similar trends with 10 and 100 μg/ml (*p* = 0.037). In the absence of antigen stimulation, cells from the Hp/EAE group gave lower counts than the Broth/EAE group (*p* = 0.003), and the response to anti-CD3/28 stimulation was also reduced by twofold (*p* = 0.001).

### *H. pylori* INFECTION WAS ASSOCIATED WITH DECREASED FREQUENCIES OF TH1 AND TH17 CELLS IN THE SPLEENS AFTER EAE INDUCTION

**FIGURE 3:**
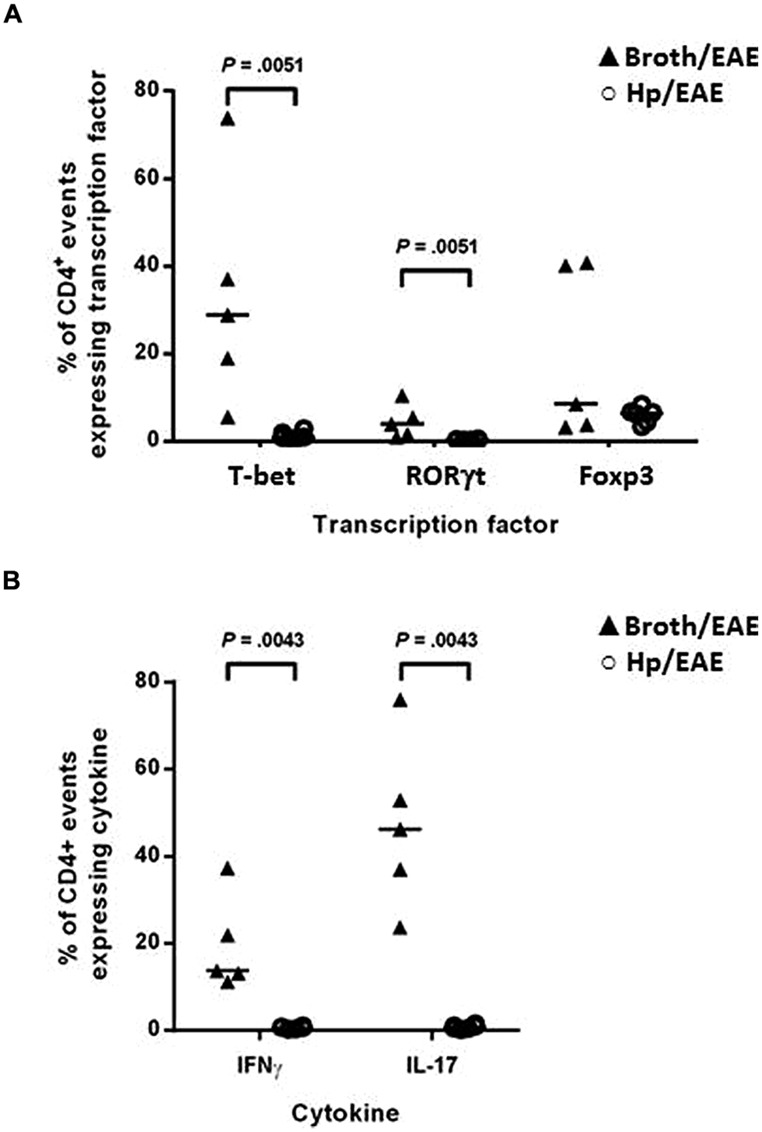
**Flow cytometry analysis of splenocytes from infected and uninfected mice with EAE.** Groups of six mice were orally inoculated with *H. pylori* (Hp/EAE) or given *Brucella* broth as a placebo (Broth/EAE). After 3 weeks EAE was induced in all mice. Spleens were disrupted, stained using fluorochrome-conjugated antibodies and analyzed using flow cytometry. Graphs show the proportion of CD4^+^ events which expressed the transcription factors T-bet, RORγt, and Foxp3 **(A)**, and the proportion of CD4^+^ events which expressed the cytokines IFNγ and IL-17 **(B)**. Medians are shown with a horizontal line. *p*-values were calculated using Mann–Whitney *U*-tests.

The proportion of CD4^+^ splenocytes expressing the signature transcription factors associated with T-helper subsets was assessed by flow cytometry. Splenocytes were isolated and immediately stained with fluorochrome-conjugated antibodies for CD4 and the transcription factors T-bet, RORγt and Foxp3. The proportions of CD4^+^ events expressing each transcription factor were determined for individual mice (**Figure 3A**). T-bet, the transcription factor expressed by Th1 cells was expressed by markedly lower proportions of CD4^+^ events in the Hp/EAE mice compared to the Broth/EAE group (medians 0.93 and 28.8%, respectively, *p* = 0.0051). Similarly, the frequency of CD4 events expressing the Th17 lineage transcription factor RORγt was also reduced (medians 0.36 and 3.88%, respectively; *p* = 0.0051). The proportion of CD4^+^ events expressing the Treg associated transcription factor Foxp3 was not statistically different between the two groups (6.40 and 8.43% in Hp/EAE and Broth/EAE mice, respectively).

In order to confirm the Th1 and Th17 data, the cytokine profiles of the CD4^+^ populations were then examined. Splenocytes were cultured in the presence of PMA/ionomycin or in medium alone for 6 h before permeabilising and staining with fluorochrome-conjugated antibodies against CD4, IFNγ and IL-17. In accordance with the transcription factor data, the proportions of CD4^+^ events expressing IFNγ and IL-17 were again lower in the Hp/EAE group. For unstimulated cultures (**Figure 3B**), 0.82% of CD4^+^ cells were IL-17^+^ in the Hp/EAE group, compared to 46.2% for the Broth/EAE mice (*p* = 0.0043). The median percentage of CD4^+^ cells expressing IFNγ was 0.58% for Hp/EAE mice compared to 13.7% for the Broth/EAE group (*p* = 0.0043). When cells were stimulated with PMA and ionomycin, the results were almost exactly the same. The frequencies of IL-17^+^ CD4^+^ cells in the Hp/EAE and Broth/EAE groups were 5.33 and 82.75%, respectively, (*p* = 0.0043), and the data for IFNγ^+^ CD4^+^ cells were 9.93 and 32.8% (*p* = 0.0043).

### *H. pylori* INFECTION WAS ASSOCIATED WITH REDUCED NUMBERS OF CD4^+^, CD8^+^, TH1, AND TH17 CELLS IN THE CNS OF MICE WITH EAE

Experimental autoimmune encephalomyelitis is characterized by infiltration of CD4^+^ and CD8^+^ cells into the CNS (Murphy et al., 2010). To determine whether *H. pylori* infection altered the pattern of infiltration observed, and if there were differences in the frequencies of Th1 and Th17 cells, CNS samples were collected from Hp/EAE and Broth/EAE mice and pooled for each group.

**FIGURE 4:**
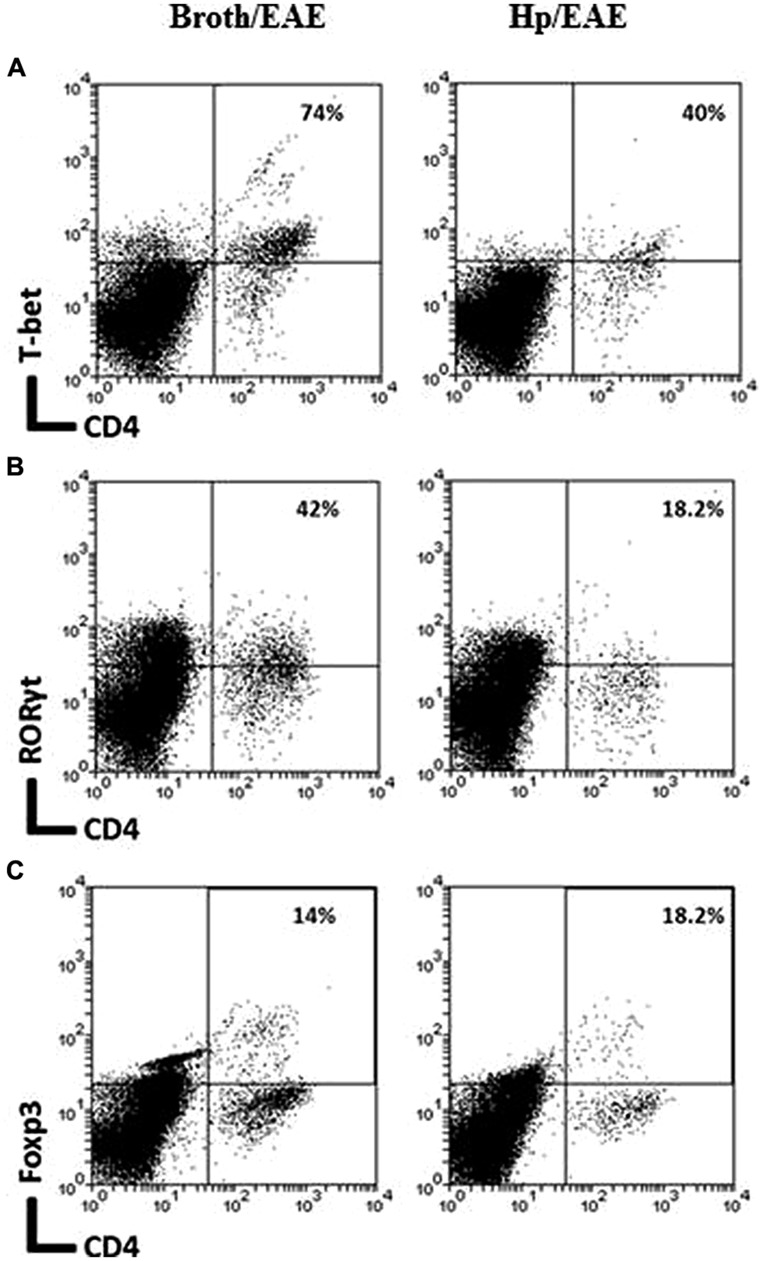
**Flow cytometry analysis of pooled cells extracted from the CNS of infected and uninfected mice with EAE.** Groups of six mice were orally inoculated with *H. pylori* (EAE/Hp) or given *Brucella* broth as a placebo (EAE/Broth). After 3 weeks EAE was induced in all mice. Samples were collected 3 weeks after EAE induction. CNS and brain tissue were pooled from each group of mice (six mice per group). Cells were extracted, stained with fluorochrome-conjugated antibodies and analyzed by flow cytometry. Lymphocyte gated dot plots showing the CD4 and T-bet **(A)**, RORγt **(B)**, and Foxp3 **(C)** staining. The percentage of CD4^+^ cells that express each marker is given in the top right hand corner.

**FIGURE 5:**
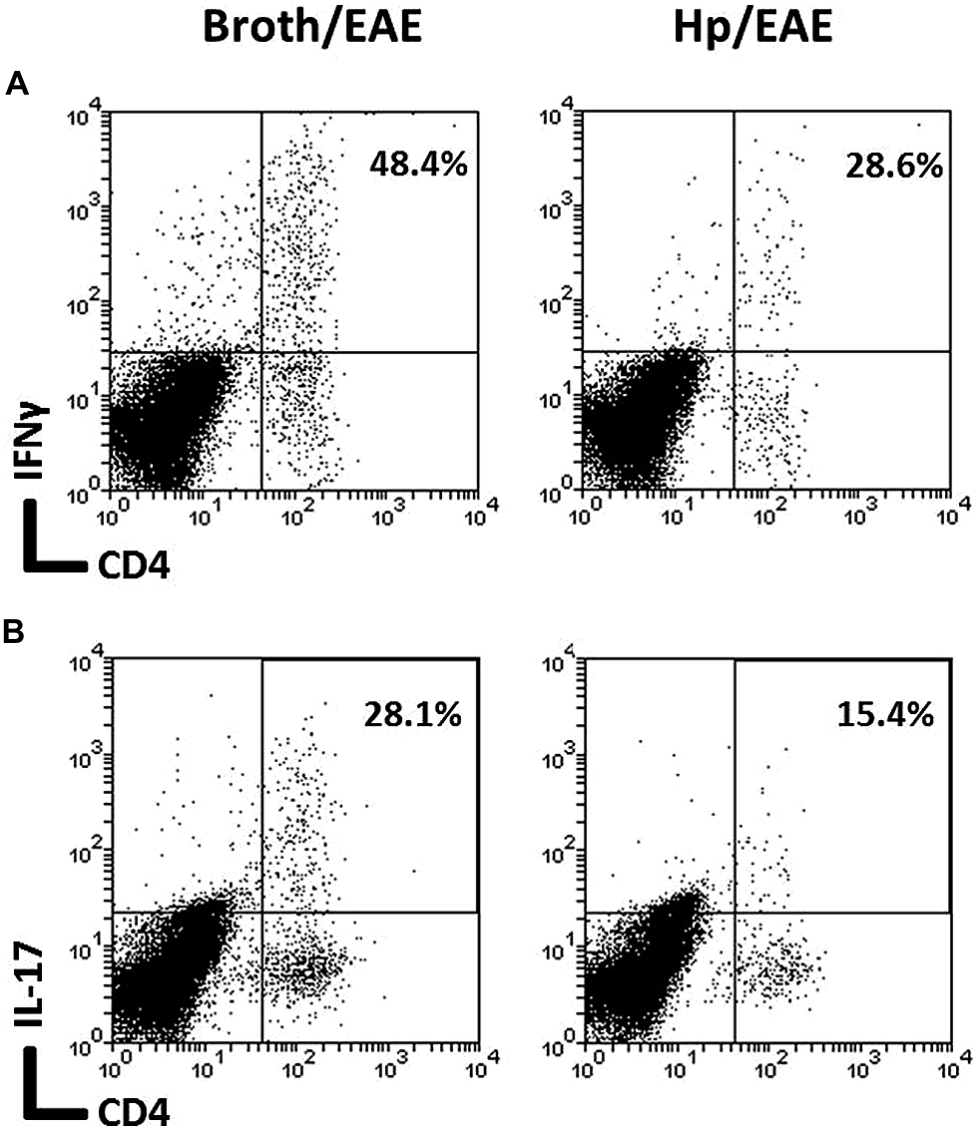
**Flow cytometry analysis of cytokine expression on pooled cells extracted from the CNS of infected and uninfected mice with EAE.** Groups of six mice were orally inoculated with *H. pylori* (EAE/Hp) or given *Brucella* broth as a placebo (EAE/Broth). After 3 weeks EAE was induced in all mice. Samples were collected 3 weeks after induction of EAE. CNS and brain tissue were pooled from each group of mice (six mice per group). Cells were extracted, stimulated with PMA and ionomycin, stained with fluorochrome-conjugated antibodies and analyzed by flow cytometry. Lymphocyte gated dot plots showing the CD4 and IFNγ **(A)** and IL-17 **(B)** staining. The percentage of CD4^+^ cells that expressed each cytokine is given in the top right hand corner.

The frequency of CD4^+^ cells was lower in the Hp/EAE group (1.1%) compared to the Broth/EAE (5.0%). The frequency of CD8^+^ cells was also lower (1.5 and 3.77%, respectively; data not shown). Transcription factor staining was used to determine the relative proportions of Th1, Th17, and Treg cells (**Figure 4**). The median proportion of T-bet^+^ cells amongst the CD4^+^ population was lower in the Hp/EAE group (40%) than the Broth/EAE mice (74%), and the proportions of RORγt^+^ Th17 cells were also reduced (18.2 and 42.0%, respectively). The frequency of Foxp3^+^ CD4^+^ cells was slightly lower in the Hp/EAE group (14%) compared to the Broth/EAE group (18.2%). The pooled cells were also stained for the cytokines IFNγ and IL-17 following PMA and ionomycin stimulation (**Figure 5**). The proportion of CD4^+^ cells expressing these cytokines was again lower in the Hp/EAE group. In the Hp/EAE sample 28.6% of CD4^+^ events were IFNγ^+^ and 15.4% were IL-17^+^, whereas in the Broth/EAE sample 48.4% of CD4^+^ events were IFNγ^+^ and 28.1% were IL-17^+^.

### *H. pylori* INFECTION DID NOT CAUSE AN INCREASE IN MARKERS OF APOPTOSIS ON CD4^+^ CELLS

**FIGURE 6:**
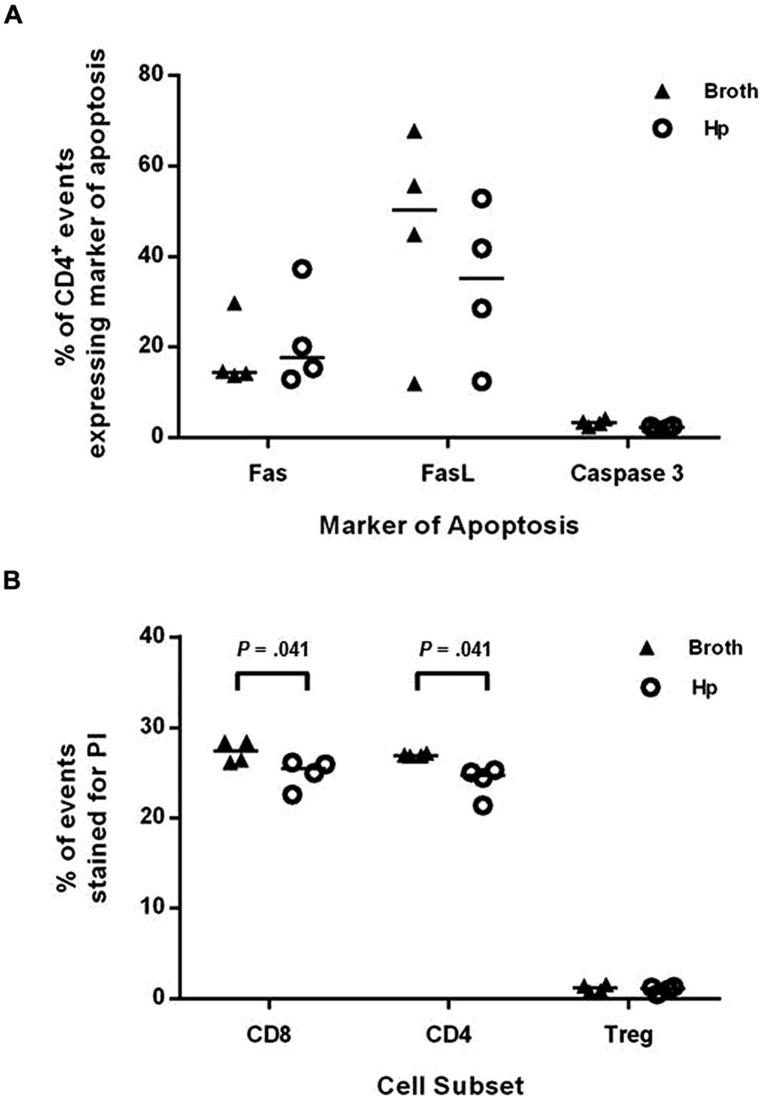
**Induction of markers of apoptosis in *H. pylori* infected mice.** Groups of four Foxp3-GFP reporter mice were infected with *H. pylori* or given placebo doses of plain broth. After 3 weeks, mice were killed and spleens were harvested. Splenocytes were stained with fluorochrome-conjugated antibodies or propidium iodide (PI) as markers for apoptosis or cell death. They were then analyzed by flow cytometry, detecting Foxp3^+^ cells as GFP^+^. The proportion of CD4^+^ events that expressed Fas **(A)**, FasL **(B),** or active caspase-3 are shown **(A)**. The proportion of CD8^+^, CD4^+^, and CD4^+^Foxp3^+^ events which were stained with PI are shown **(B)**. Medians are depicted as horizontal lines for each group. *p*-values were calculated using Mann–Whitney *U*-tests.

*Helicobacter pylori* infection has previously been shown to cause increased T cell apoptosis *in vivo*, via the induction of FasL (Wang et al., 2001). To investigate if this may be a mechanism for reducing the numbers of Th1 and Th17 cells in the spleen and CNS, groups of four Foxp3-GFP reporter mice were infected with *H. pylori* or given placebo broth doses. After 3 weeks (when EAE-induction would have commenced), splenocytes were collected, stained for a number of markers of apoptosis and cell death, and assessed by flow cytometry (**Figure 6**). The proportion of CD4^+^ events expressing either Fas, FasL, or active caspase-3 was not significantly altered during *H. pylori* infection. The proportion of CD4^+^ and CD8^+^ events which took up PI, a membrane-impermeable dye taken up by dead cells, was significantly lower in the infected group but these differences were very small and are unlikely to be biologically relevant. For CD4^+^ events, 24.7 and 26.9% were PI^+^ amongst infected and uninfected mice, respectively, (*p* = 0.041). For CD8^+^ events, 25.5 and 27.4% were PI^+^ amongst infected and uninfected mice (*p* = 0.041). There was no difference in the proportion of PI^+^ events amongst gated CD4^+^ Foxp3^+^(GFP^+^) cells.

## DISCUSSION

This study examined the direct protective role of *H. pylori* infection on development of EAE for the first time. The results show that prior infection with *H. pylori* altered the immune response to EAE induction, and had a small but significant protective effect on the clinical outcome of EAE.

Experimental autoimmune encephalomyelitis is the most commonly used model for investigating human MS (Constantinescu et al., 2011). In this study EAE was induced by immunization with MOG_35-55_ peptide in the C57BL/6 mouse strain, which leads to an autoimmune response mimicking aspects of chronic MS (Constantinescu et al., 2011; ’t Hart et al., 2011). The C57BL/6 mouse is the most commonly used for investigating *H. pylori* infection and immunity, including the mechanisms of protection against allergy and colitis (Arnold et al., 2011; Higgins et al., 2011). We found that maximal clinical scores of EAE were significantly reduced in mice with an established *H. pylori* infection. Infected mice on average had milder hind-limb paralysis. The time to EAE onset was not altered by *H. pylori* infection, however, suggesting that the infection did not interfere with the induction of EAE, but inhibited the development and severity of motor deficits. This observation correlates with a clinical study which found that MS patients with positive *H. pylori* serology tests had lower disability scores than seronegative patients (Mohebi et al., 2013). Infiltration of leukocytes into the CNS has been shown to correlate with lesion load within the spinal cord of EAE mice (Baker et al., 2011). Those infected with *H. pylori* had markedly reduced numbers of Th1 and Th17 cells in both the CNS and spleen. Infiltration of these effector T cells into the CNS is a major marker of disease severity in EAE (Murphy et al., 2010; Lovett-Racke et al., 2011), confirming the importance of *H. pylori* infection in altering the pathogenic immune response in immune-mediated inflammatory demyelination. Further studies are required to more clearly establish the role of Th1 and Th17 cells in MS lesions (Lovett-Racke et al., 2011).

Myelin-specific Th1 and Th17 cells are found in the CNS of EAE mice and MS patients, and both are thought to play a role in disease pathogenesis (Lovett-Racke et al., 2011). Evidence suggests that T-bet^+^ T cells are of particular importance in the generation of CNS inflammation and demyelinating lesions. In EAE, both IFNγ^+^ and IL-17^+^ cells may express T-bet (Yang et al., 2009; Grifka-Walk et al., 2013). In line with the fact that CNS-infiltrating Th17 cells may acquire Th1 characteristics in EAE (Lovett-Racke et al., 2011; Grifka-Walk et al., 2013), we found that T-bet^+^ cells were more prevalent than RORγt^+^ cells in the spleen and CNS. Interestingly, we have shown that the *H. pylori-*infected EAE mice had 30-fold fewer T-bet^+^ cells in the spleen than Broth/EAE mice. The reduction in RORγt^+^ cells was 10-fold. We anticipate that these dramatic changes in the immune cell populations are responsible for the difference in EAE clinical scores. Although many studies have focused on the role of CD4^+^ cell populations in EAE and MS, CD8^+^ cells are also important (Saxena et al., 2011). Antigen-specific CD8^+^ cells infiltrate the CNS, causing inflammatory lesions in the optic nerve, brain and spinal cord, with focal loss of oligodendrocytes and axonal damage (Saxena et al., 2011). In our experiments we found a twofold reduction in CNS CD8^+^ cells from infected EAE mice compared to the Broth/EAE group. This difference was not as large as for the CD4^+^ population (fivefold reduction), but it is likely to have an impact on the level of inflammatory damage and thus contribute to reduced EAE severity in infected mice.

Th1 and Th17 cells are also associated with the gastric mucosal immune response elicited by *H. pylori* infection (Gray et al., 2013). It is therefore interesting that infected mice had reduced numbers of these cells in the spleen and CNS after EAE induction. The difference in numbers of CD4^+^ cells in the CNS did not appear to be linked with increased levels of T cell apoptosis, however, this was not explored in mice after EAE induction. Our initial hypothesis was that, similar to *H. pylori-*mediated protection from asthma (Arnold et al., 2011), reduced EAE severity could be due to the induction of an enhanced Foxp3^+^ Treg population. Such cells might act by suppressing the induction and activity of MOG-specific Th1 and Th17 effector cells. Whilst total numbers of CD4^+^ T cells were decreased in the CNS of *H. pylori* infected mice, *H. pylori* infection was not associated with increased proportions of Tregs amongst them in either the spleen or the CNS. In the present study, we limited our quantification of Tregs to Foxp3^+^ cells, and this work must now be expanded to examine other Treg populations. *H. pylori-*induced Tregs tend to act via secretion of the suppressive cytokine IL-10 (Robinson et al., 2008; Arnold et al., 2012), and the IL-10-secreting Tr1 type of Tregs are Foxp3^-^ (Roncarolo et al., 2014). It has recently been shown that a subset of FoxA1^+^, Foxp3^-^ Tregs are protective against EAE, and these are also present in humans (Liu et al., 2014).

A number of other potential mechanisms will need to be investigated in the future. In patients, *H. pylori* infection is associated with alterations in the profile of homing receptors expressed by peripheral blood T cells, directing their migration toward the inflamed gastric mucosa (Lundgren et al., 2005). We previously showed that *H. pylori* infection results in increased proportions of human peripheral blood Tregs that express the chemokine receptor CCR6 (Cook et al., 2014). CCR6 has been implicated in EAE progression, with one study showing that CCR6 deficient mice develop less severe disease (Liston et al., 2009) and another concluding that they are less able to control EAE when it develops (Elhofy et al., 2009). It has been suggested that CCR6 is also important in moderating the balance between Tregs and Th17 cells (Comerford et al., 2010). The infection may therefore alter the expression of chemokine receptors and integrins by T-effector or regulatory T cells, resulting in fewer T cells entering the CNS and thus inhibiting EAE development.

In allergy studies, *H. pylori* infection has been shown to stimulate the differentiation of tolerogenic DC populations, which provide protection against the development of allergic asthma (Oertli and Muller, 2012). Interestingly, the peak of severity in EAE has previously been shown to correlate with DC recruitment to the CNS (Sagar et al., 2012). Given that this study showed that *H. pylori* infection altered the clinical severity at the peak of EAE clinical scores, the involvement of DCs should also be further investigated.

This study provides the first suggestion that *H. pylori* infection reduces the severity of EAE in mice, which has important implications. We and others demonstrated a negative association between *H. pylori* infection and Western-type (Kira et al., 1996) MS in patients (Wender, 2003; Li et al., 2007; Mohebi et al., 2013; Yoshimura et al., 2014), however, this approach could not establish a causal relationship. *H. pylori* may merely be a marker for other protective factors. Atherton and Blaser (2009) put forward two hypotheses to explain the mechanisms by which *H. pylori* and allergy could be negatively associated. They suggested that *H. pylori* infection either directly alters the immune response, leading to decreased risk of allergy, or that the presence of other factors such as commensal bacteria and parasite infections dampen the immune response, leading to both increased *H. pylori* infection and decreased risk of allergic disease (Atherton and Blaser, 2009). There is evidence from the Mongolian gerbil model that *H. pylori* infection causes alterations in the microbiota of the inflamed stomach and duodenum (Yin et al., 2011). A recent long-term colonization study in gerbils, using a pathogenic *H. pylori* strain, showed that there was a change in the microbiota of the large intestine (Heimesaat et al., 2014). Since the gut microbiota is known to have an impact on EAE (Berer et al., 2011; Lee et al., 2011), it remains a possibility that the protective effects of *H. pylori* are mediated indirectly via manipulation of the flora.

In conclusion, our studies provide strong evidence that *H. pylori* infection exerts an impact on MS and EAE. Further mechanistic animal model experiments and longitudinal clinical studies are now necessary to fully evaluate the effects of *H. pylori* on development of conventional MS.

## AUTHOR CONTRIBUTIONS

Karen Robinson, Bruno Gran, Cris S. Constantinescu, Katherine W. Cook, Kate O’Brien, Khiyam Hussain, and James Crook conceived and planned the study. Karen Robinson, Bruno Gran, Katherine W. Cook, Kate O’Brien, Khiyam Hussain, Manjit Braitch, Huner Kareem, and James Crooks carried out the experimental work. Katherine W. Cook, James Crooks, Manjit Braitch, Huner Kareem, Khiyam Hussain, Bruno Gran, and Karen Robinson analyzed the data. The manuscript was prepared by Karen Robinson, Katherine W. Cook, James Crooks, and Bruno Gran. All authors revised the manuscript, approved the final version submitted, and agree to be accountable for all aspects of the work in ensuring that questions related to the accuracy or integrity of any part of the work are appropriately investigated and resolved.

## Conflict of Interest Statement

The authors declare that the research was conducted in the absence of any commercial or financial relationships that could be construed as a potential conflict of interest.
